# Time until symptoms and design-related associations in Alzheimer’s disease clinical progression analyses

**DOI:** 10.64898/2026.05.10.26352825

**Published:** 2026-05-13

**Authors:** Kellen K. Petersen, Yan Li, Suzanne E. Schindler

## Abstract

**Introduction:**

Studies of the risk and timing of symptomatic Alzheimer’s disease (AD) in cognitively unimpaired individuals are challenging due to the relatively small number of clinical progressors and limited clinical follow-up, which can lead to design-related associations. Clock models can be used to anchor the timing of events to biological events such as biomarker positivity. We hypothesized that estimated age at plasma %p-tau217 positivity based on clock models is less affected by design-related associations as compared to baseline age.

**Methods:**

Data from the Knight Alzheimer Disease Research Center (Knight ADRC) and Alzheimer’s Disease Neuroimaging Initiative (ADNI) were analyzed. Age at %p-tau217 positivity was estimated using two clock model approaches, TIRA and SILA. The C-index of estimated age at plasma %p-tau217 positivity and age at the baseline plasma sample (baseline age) for ranking age of AD symptom onset was evaluated in initially cognitively unimpaired individuals, including progressors and non-progressors. In progressor sub-cohorts, baseline age and time from %p-tau217 positivity to baseline were associated with time from baseline until symptom onset; baseline age and estimated age at %p-tau217 positivity were associated with age at symptom onset. Commonality analyses partitioned the variance unique to each predictor and shared between predictors. Randomization analyses evaluated whether observed associations exceeded those expected by chance.

**Results:**

Estimated age at %p-tau217 positivity enabled analyses of a greater number of progressors in the research cohorts, which did not have plasma %p-tau217 data from every clinical assessment. The estimated age at %p-tau217 positivity had a higher C-index than baseline age for ordering the likelihood of AD symptom onset when all follow-up was considered; when follow-up was truncated, the C-index for estimated age at %p-tau217 positivity remained stable while the C-index for baseline age became inflated. In progressors, estimated age at %p-tau217 positivity contributed unique variance beyond baseline age in associations with age at symptom onset. Randomization analyses in the larger Knight ADRC found that associations between clock-derived measures and time from baseline until symptom onset and age at symptom onset exceeded the permuted null distribution, with some mixed results in the smaller ADNI cohort.

**Conclusions:**

Compared to baseline age, the biologically-anchored estimated age at %p-tau217 positivity is less susceptible to design-related associations and incrementally improves prediction of age at symptom onset in analyses conditional on progression.

## Full Text Availability

The license terms selected by the author(s) for this preprint version do not permit archiving in PMC. The full text is available from the preprint server.

